# A patient-derived ccRCC model that retains native stromal features to assess fibrosis-driven personalized therapeutic response

**DOI:** 10.1038/s41598-026-50508-z

**Published:** 2026-04-28

**Authors:** Alexis Perreault, Kelly Harper, Martine Charbonneau, Karine Brochu-Gaudreau, Robert Sabbagh, Patrick O. Richard, Nadia Ekindi-Ndongo, Claudio Jeldres, Claire M. Dubois

**Affiliations:** 1 Department of Immunology and Cell Biology, Université de Sherbrooke, Sherbrooke, QC J1H 5N4 Canada; 2 Division of Urology, Department of Surgery, Université de Sherbrooke, Sherbrooke, QC J1H 5N4 Canada; 3 Department of Pathology, Université de Sherbrooke, Sherbrooke, QC J1H 5N4 Canada

**Keywords:** Cancer, Cell biology, Oncology

## Abstract

Clear cell renal cell carcinoma (ccRCC) is characterized by a complex tumor microenvironment, in which stromal components such as collagen and cancer-associated fibroblasts (CAF) play a pivotal role in therapeutic resistance. To investigate the contribution of fibrosis to interpatient variability in treatment response, we developed a patient-derived xenograft (PDX) model using the chick chorioallantoic membrane (CAM) *ex ovo* assay. This model preserves key stromal features of the original tumors, including collagen types I, III and VII, fibronectin, lysyl oxidases (LOX), and CAF markers such as fibroblast activation protein (FAP) and α-smooth muscle actin (α-SMA). Transcriptomic analyses confirm the conservation of gene expression profiles related to stromal architecture, extracellular matrix composition, and cellular metabolism within the xenografts. *In vivo* assays across multiple PDXs reveal heterogeneous responses to antifibrotic agents, which correlate with the collagen content of the original tumors. Notably, antifibrotic pretreatment enhances therapeutic efficacy in xenografts derived from tumors with collagen levels ranging from 5 to 25%. This model provides a physiologically relevant platform to assess fibrosis-driven therapeutic responses in ccRCC and supports the integration of stromal profiling into personalized treatment strategies.

**Supplementary Information:**

The online version contains supplementary material available at 10.1038/s41598-026-50508-z.

## Introduction

Renal cell carcinoma (RCC) represents 2-3% of all adult malignancy diagnoses and deaths, with a continual global rise in incidence over the past decades and a projected ongoing increase^1^. The most aggressive subtype, clear cell RCC (ccRCC), accounts for 70-75% of all diagnosed RCC^2,3^. While localized ccRCC is commonly treated through partial or radical nephrectomy, 20-40% of patients experience recurrence and metastasis following tumor removal^4^. More recently, the management of advanced ccRCC has been revolutionized by immune checkpoint inhibitors^5^. However, immunotherapy alone is often insufficient to achieve durable response and is now combined with targeted therapies such as tyrosine kinase inhibitors (TKIs) and mammalian target of rapamycin (mTOR) inhibitors^5^. Moreover, TKI monotherapy remains a first-line option for patients who cannot receive immunotherapy^6^. Despite significant progress in the development of targeted therapies and immunotherapies for advanced ccRCC, the majority of patients are refractory to treatment and will eventually experience progression of the disease^7^.

Recent studies have highlighted the role of extracellular matrix (ECM) in cancer development and resistance to treatment^8,9^. Indeed, fibrotic stroma accumulates in the tumor microenvironment due to the activation of CAFs, which in turn secrete factors known to promote immune exhaustion, tumor growth, invasion, and metastasis^10^. Fibrosis can also hinder tumor vascularization through mechanical compression of blood vessels, thus restricting the biodistribution of therapeutic agents and immune infiltration^11^. In ccRCC, the extent of intratumoral fibrosis has been associated with poor prognostic factors, such as tumor grade, intratumoral necrosis, and lymphovascular invasion^12^. In addition, the expression of numerous collagen subtypes (e.g., types I, V, VI, and VII) is correlated with negative patient outcomes in ccRCC^11^. In line with the role of ECM in cancer progression and treatment, efforts are being made to repurpose drugs approved for fibrotic diseases such as nintedanib and pirfenidone, used for idiopathic pulmonary fibrosis (IPF), and to investigate their efficacy in malignant cancers^13^. Notably, the efficacity of combination therapies targeting both cancer-associated fibrosis and tumor cells has been demonstrated *in vitro* and in preclinical and clinical studies for several types of cancer, including lung, breast, and pancreatic cancers^14–16^. However, this possibility has not yet been addressed in the context of ccRCC.

To investigate tumor-associated fibrosis in human tissues, accurate models that maintain the structural integrity and key components of the fibrotic microenvironment are necessary. We previously established an *in vivo* model that consisted of cultivating ccRCC PDXs on the CAM of chick embryos (CAM-ccRCC model)^17^. The very high engraftment rate of 100% per patient and 94% for individual implanted tumor fragments obtained with this model allows for many technical replicates to be performed, features that cannot be replicated using other models^17^. Moreover, this model is cost-effective, complies with the 3Rs principles for humane animal research^18^ and allows for experiments to be performed within a 7-day experimental timeframe, including evaluating the response to targeted therapy^17^. Recently, the utility of the CAM model to study fibrosis was supported by a study that demonstrated that lung tissue derived from IPF patients cultivated on the CAM maintained their integrity and IPF phenotype^19^. In this model, treatment with the FDA- and Health Canada-approved drug, nintedanib, as well as other antifibrotics in clinical development such as setogepram, significantly modulated fibrosis-associated markers in IPF lung tissue xenografts, suggesting that the CAM model could be an appropriate approach to study the response to antifibrotics in human ccRCC tumors.

In this study, we established that the CAM-ccRCC model is a robust tool for studying the fibrotic microenvironment of ccRCC patient tumors. Xenografts cultivated on CAM retained the collagen levels and transcriptomic profiles of the original tumors, along with fibrosis markers associated with poor prognosis in ccRCC patients. We also showed that fibrosis can be effectively modulated in this model, particularly with setogepram. Notably, pretreatment of ccRCC-derived xenografts with this antifibrotic significantly potentiated the therapeutic efficacy of the TKI drug sunitinib. Finally, we identified an intratumoral collagen content in the range of 5-25% as a predictive biomarker of treatment response. Collectively, these findings highlight the feasibility of using the CAM-ccRCC model as a drug discovery and personalized treatment platform for hard-to-treat ccRCC tumors.

## Methods

### ccRCC tissue collection and preparation

ccRCC patient tumors were obtained from patients undergoing partial or radical nephrectomy at the Centre Hospitalier Universitaire de Sherbrooke (CHUS) (2018-2024). Inclusion criteria were patients with diagnosed ccRCC that were at least 18 years of age. The clinical characteristics of the Sherbrooke ccRCC cohort, including subject demographics are presented in Table 1. The study was conducted according to the guidelines of the Declaration of Helsinki under a protocol approved by the Research Ethics Board of the CIUSSS de l’Estrie-CHUS (#2017-1524). Informed consent was obtained from all patients. A pathologist established the pathological diagnosis, grade and stage according to the WHO 2016 (2018-2022) or the WHO 2022 (2022-2024) grading system^2,3^ and the 8^th^ AJCC TNM staging system^20^. Fresh tumor tissue specimens were collected in the Department of Pathology of the Université de Sherbrooke and engraftment on CAM was performed within 24 hours after resection. When specimens could not be implanted within 24 hours, tissues were frozen shortly after resection for subsequent use. Necrotic tissue was carefully removed using a surgical blade prior to engraftment. Sections of tumor tissue were also snapfrozen and kept at -80ºC or fixed in formalin for 24 hours and embedded in paraffin for histopathological analysis.

**Table 1:** Clinical characteristics of the Sherbrooke ccRCC cohort.

**Clinical characteristic**	**Number**	**%**
**Sex**		
Male	27	77
Female	8	23
**Age, years**		
Mean ± SEM	63 ± 1.5	
Range	40 - 80	
**Histology**		
Clear cell renal cell carcinoma	35	100
**Grade (WHO**^**1**^**)**		
1	0	0
2	17	48
3	15	43
4	3	9
**Stage (TNM**^**2**^**)**		
1	14	40
2	0	0
3	20	57
4	1	3

^1^WHO: World Health Organization

^2^TNM: Tumor (T), Nodes (N), Metastasis (M).

### Reagents

Purified nintedanib and setogepram were provided by Liminal R&D BioSciences Inc (Laval, QC, Canada). Sunitinib (cat#S-8803) was from LC-Laboratories (Woburn, MA, USA). Human-specific anti-pro-collagen type I (cat#ab64409), anti-collagen type III (cat#ab7778), anti-FAP (cat#ab207178) and anti-LOXL2 (cat#96233) antibodies were from Abcam Inc (Toronto, ON, Canada). Anti-collagen type VII (cat#HPA042420) and anti-fibronectin (cat#MAB1940) were from MilliporeSigma (Oakville, ON, Canada). Anti-cleaved caspase 3 (cat#9664S) was from Cell Signaling (Boston, MA, USA) and anti-CAIX (cat#sc-25599) was from Santa Cruz (Dallas, TX, USA). Anti-α-SMA (cat#M0851), anti-CD31 (cat#M0823), as well as mouse IgG (cat#X0931) and rabbit IgG (cat#X0903) isotype controls were from Agilent Dako (Santa Clara, CA, USA). Biotin-SP conjugated secondary antibodies biotin-SP goat anti-rabbit IgG (cat#111-065-045) and goat anti-mouse IgG (cat#115-065-062) were from Jackson ImmunoResearch Laboratories Inc (West Grove, PA, USA). Biotinylated goat anti-rat IgG (cat#BP-9400) was from Vector Laboratories (Burlingame, CA, USA).

### CAM assay

Fertilized white leghorn chicken eggs used for the *in vivo* ccRCC PDX study were obtained from the Public Health Agency of Canada (Nepean, ON) or the Couvoir Boire et Frères Inc. (Wickham, QC, Canada). The project was approved by the Ethics Committee of Animal Research of the Université de Sherbrooke (Protocol #054-17). All experimental procedures involving chick embryos were conducted in accordance with the regulations of the Canadian Council on Animal Care (CCAC) guidelines. When applicable to the CAM assay, the reporting of animal experiments complied with the Animal Research: Reporting of *In Vivo* Experiments (ARRIVE) guidelines. CAM assays were performed using the *ex-ovo* model to facilitate access to CAM vasculature, as previously described^17^ with the following modifications. At embryonic developmental day (EDD) 9, CAMs were randomized, and freshly resected specimens were cut into tissue fragments with a diameter between 1 and 2 mm and implanted directly onto the CAMs in a 1:1 mixture of Matrigel (VWR, cat# CACB354234) and McCoy’s 5A culture medium with 10% FBS for a total volume of 10 μL. For drug testing, nintedanib and setogepram were injected into the CAM vasculature (10-12 animals per group) at human-equivalent doses, as indicated in the figure legends, 2 days after the implantation of tumor fragments. For combination therapy, setogepram was administered topically onto the xenografts at EDDs 9 and 10 at a concentration of 500 µM in a volume of 20 µL, prior to the injection of 200 µg of setogepram and 10 µg of sunitinib at EDD 11. On EDD 16, chick embryos were euthanized by decapitation in accordance with CCAC guidelines and were examined macroscopically for indications of drug toxicity including mortality rate, head and body development, and extra-embryonic structures (CAM and vasculature). All treatments used in this study had no effect on any of these aspects. Vascularized tumor masses that exhibited no discernible signs of necrosis were considered successfully engrafted and were removed from the CAM on EDD16. Images of extracted xenografts were taken with the LEICA S9i microscope, and volumes were blindly measured using LAS X software (version 5.0.3.24880, https://www.leica-microsystems.com, Leica Microsystems, Concord, ON, Canada) and calculated using the length*width^2^/2 formula^19^. A portion of the xenografts or original tissue was snap frozen and kept at -80ºC for gene expression analysis and the other portion was fixed in formalin for 24 hours and subsequently embedded in paraffin for histopathological analysis.

### Histology, immunostaining, and quantification method

Paraffin-embedded tissue sections from original renal tissues or CAM xenografts were deparaffinized, rehydrated and stained. Immunohistochemical staining was performed on serial tissue sections using the standard streptavidin-biotin immunoperoxidase complex technique. In brief, primary biotin-SP conjugated secondary antibodies of appropriate specificity were used for antigen detection. The incubation step involving streptavidin-biotin peroxidase complex (Jackson ImmunoResearch Laboratories Inc., cat#016-030-084) was then conducted prior to the detection process with diaminobenzidine (MilliporeSigma, cat#D4168) and subsequent counter staining with Harris Hematoxylin (Epredia, Kalamazoo, MI, USA). Masson’s trichrome (ScyTek Laboratories Inc., Logan, UT, USA, cat#TRM-1-IFU) was used to detect collagen deposition and performed following the manufacturer’s instructions. Stained tissues were scanned at 40X magnification using the Hamamatsu NanoZoomer 2.0 RS slide scanner (Hamamatsu Photonics, Bridgewater, NJ, USA) for further analysis.

The quantification of the percentage of positive area for collagen or specific markers of tumors and xenografts stained with Masson’s trichrome or immunohistochemically stained was done using ImageJ software (version 1.53, http://imagej.net/ij, National Institutes of Health, Bethesda, MD, USA), as previously described^21^. The Color Deconvolution ImageJ plugin was employed to extract the regions of the xenografts that were stained blue for collagen, or brown for specific markers. This method enables the quantification of the area occupied by these regions and of the total area of the xenografts (in pixels). The percentage of positive area was then calculated using the following formula: (positive area/ total area of the xenograft) *100. Interobserver variability was assessed using the intraclass correlation coefficient (ICC), based on a two-way random effects model for absolute agreement between single ratings. The resulting ICC(A,1) was 0.99 (95% CI: 0.0967-0.997), and the F-test confirmed that this level of agreement was highly significant (F(15, 10.6)=250, p=2.42x10^-11^).

A segmented linear regression was performed to evaluate changes in the relationship between the percentage of collagen positive area in original tumors and the percentage of collagen inhibition of collagen content in CAM xenografts, with a fixed breakpoint at x=25, reflecting a clear shift in slope. Models were fit using ordinary least squares, with an interaction term allowing for distinct slopes and intercepts before and after the breakpoint. Improvement in model fit compared to a simple linear regression was assessed via F-tests for nested models using the ANOVA function in the R software (version 4.5.0, http://www.R-project.org, The R Foundation for Statistical Computing, Vienna, Austria).

### RNA preparation and sequencing

Tissue xenografts extracted from the CAMs were homogenized using a mortar and pestle in liquid nitrogen. Total RNA was isolated using the RNeasy® Mini Kit (Qiagen, Mississauga, ON, Canada, cat#74106) in accordance with the manufacturer’s instructions. Each RNA-seq library was generated from 500 ng of total RNA free of genomic DNA using the NEBNext® Ultra™ II Directional RNA Library Prep Kit for Illumina (New England Biolabs, Whitby, ON, Canada, cat#E7760S), following the manufacturer’s protocol in combination with the NEBNext Poly(A) mRNA Magnetic Isolation Module (New England Biolabs, cat#E7490). The resulting libraries underwent a total of 11 amplification cycles and were purified using 0.9X Ampure XP beads (Beckman Boulter, Mississauga, ON, Canada, cat#A63881). Quality and size of fragments were assessed using an Agilent 2100 Bioanalyzer (Agilent Technologies, Mississauga, ON, Canada). Libraries were then quantified with a Qubit fluorometer (Thermo Fisher Scientific, Mississauga, ON, Canada, cat#Q32866), pooled at equimolar concentrations, diluted to 1.8 pM, and sequenced on an Illumina NextSeq 500 (illumina, San Diego, CA, USA, cat#SY-515-1001) using the NextSeq 500/550 High Output Kit v2.5 (75 cycles) (illumina, cat#20024907) with a 2 × 43 bp paired-end run configuration. RNA preparation and sequencing were performed by the Plateforme RNomique de l’Université de Sherbrooke.

### RNA-seq data processing and normalization

Reads were trimmed using Trimmomatic, and their quality was assessed using FastQC^22^. Kallisto was used to pseudo-align the reads to the transcriptome and quantify transcript abundance^23^. A combined transcriptome reference was generated using gffread from Ensembl v109 annotations and genome file for the human genome (GRCh38)^24^. Transcript-level abundances were summarized to gene-level counts using the tximport package^25^. The datasets generated and analysed during the current study are available in the Gene Expression Omnibus (GEO) repository (GSE315653). Count data were rounded and transformed using the variance-stabilizing transformation (VST) function from the DESeq2 package (v1.40.2) to stabilize variance across samples^26^. To focus the analysis on human samples, only original tumor (Tumor) and CAM xenograft (PDX) samples were retained for the VST and the 2500 most variable genes were selected based on variance across these samples to limit the noise effect of expression data. The “Host” sample (CAM) was next appended to the VST-normalized matrix. The combined matrix (Tumor, PDX, and Host) was then scaled to Z-scores using the mean and standard deviation calculated across “Tumor” and “PDX” samples only to ensured that clustering and PCA were not influenced by the host-specific expression profile. RNA-Seq data processing and normalization were performed by the Plateforme RNomique de l’Université de Sherbrooke.

### Principal component analysis, unsupervised clustering and heatmap visualization

Principal component analysis (PCA) was performed using the scaled expression matrix of the 2500 most variable genes and conducted using the prcomp function in R^27^. The first two principal components were extracted and visualized using ggplot2. Hierarchical clustering of genes was performed using Ward’s method (ward.D2) based on euclidean distances of Z-scored gene expression values and genes were divided into 10 clusters using the cutree function^28^. The heatmap was generated using the pheatmap package, displaying the scaled expression of the 2500 selected genes across all samples.

### Gene ontology enrichment analysis

For each of the 10 gene clusters obtained from hierarchical clustering, Gene Ontology (GO) enrichment analysis was performed using the clusterProfiler package (v4.10.1) and the org.Hs.eg.db database after gene symbols were converted to Entrez IDs via the bitr function^29^. GO enrichment was conducted for each Biological Process (BP) using a Benjamini-Hochberg adjusted p-value threshold of 0.05.

### TCGA data analysis

Gene expression data were obtained from publicly available The Cancer Genome Atlas (TCGA) Illumina HiSeq RNA-Seq (RSEM normalized) datasets using TCGA Data Portal (KIRC-TCGA available on the cbioportal.org database)^30^. The clear cell renal cell carcinoma (KIRC) cohort comprised 533 patients. Affymetrix gene expression RSEM results and associated overall survival data from ccRCC patients were used to assess the correlation between the expression of various genes and prognosis. Hazard ratios were calculated using Logrank test based on Kaplan-Meier survival curves of overall survival using patients in the highest and lowest quartiles of expression of the presented genes.

### Statistical analyses

The data are presented as the median or mean, as indicated in the figure legends, and the error bars indicate the standard error of mean or median (SEM), when appropriate. The statistical analyses were conducted using Prism (version 11.0.0, https://www.graphpad.com, GraphPad Software, Boston, MA, USA) and R softwares. The statistical tests used to assess statistical significance are indicated in figure legends, with a P value set at <0.05.

## Results

### The original fibrotic microenvironment of ccRCC tumors is preserved in the CAM model

**Fig. 1:**
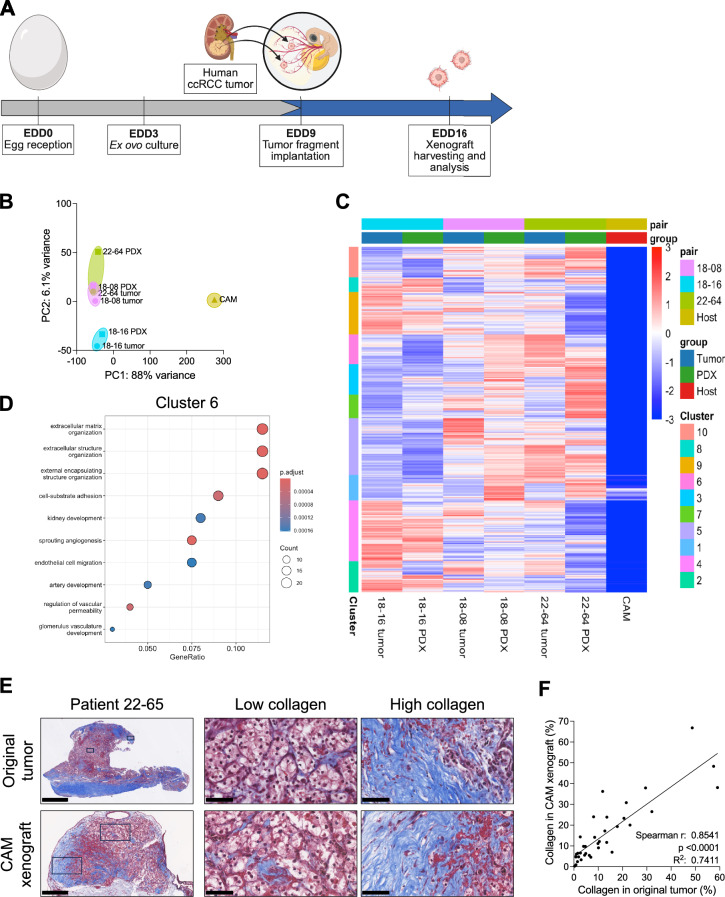
Key ccRCC and fibrosis-related signatures found in tumors from ccRCC patients are maintained on CAM. **A**) Experimental protocol for the development of the CAM-ccRCC model. Created in BioRender. Dubois, C. (2026) https://BioRender.com/fl3wtgg. (**B**) PCA analysis of the patient’s original tumor compared to the corresponding CAM xenograft (original tumor and associated PDXs from 3 patients) and a control CAM sample. Tumor and corresponding clusters are highlighted with colored ovals. **C**) Heatmap of the top 2500 most variable genes (z-score scaled).** D**) GO enrichment dot plot for genes in cluster 6. Adjusted p-values <0.05 using Benjamini-Hochberg method for False Discovery Rate correction. **E)** Representative Masson’s trichrome staining images of the patient’s original tumor compared to the corresponding CAM xenograft showing collagen deposition patterns (collagen in blue, cytoplasm in red, nuclei in black). Scale=1mm (patient tumor), 250µm (CAM xenograft) and 50µm for higher magnifications. **F)** Correlation between the percentage collagen positive areas in the original and corresponding CAM xenografted tumors. Each dot represents the collagen positive area for one patient (original tumor and associated PDXs from 35 patients), Spearman’s correlation test.

The CAM-ccRCC model, generated by implanting patient-derived tumor fragments on the CAM for 7 days, was previously shown to preserve tumor integrity and the expression of markers enriched in ccRCC, including cytokeratin 18, vimentin, and carbonic anhydrase 9 (CAIX, hypoxia)^17^. To evaluate its potential to study tumor-associated fibrosis, we now examined whether patient-derived ccRCC xenografts retained the fibrotic microenvironment of the original tumor **(**Fig. 1A). The clinical characteristics of patients who underwent nephrectomy at the Centre Hospitalier Universitaire de Sherbrooke (Sherbrooke ccRCC cohort) are presented in Table 1. As a first step, we performed RNA-sequencing to confirm that the gene expression profiles of ccRCC tumors were maintained in their corresponding CAM xenografts. Principal component analysis (PCA) based on the top 2500 most variable genes in the samples revealed that original tumors from 3 ccRCC patients and their corresponding CAM xenografts each formed clusters, which diverged greatly from the control CAM sample (Fig. 1B). The first two principal components, PC1 and PC2, accounted for 88% and 6.1% of the total variance, respectively. Genes were grouped into 10 clusters using hierarchical clustering and a heatmap displaying the z-scored expression level of the 2,500 most highly modulated genes was generated to visualize the gene expression profile of the samples. The findings indicated that a significant number of gene expression clusters were conserved between the original tumors and the corresponding CAM xenografts (Fig. 1C). Interestingly, the gene ontology enrichment analysis identified several enriched biological processes that were relevant to renal tumor xenograft development, including “epithelial cell proliferation” (cluster 1), “renal system development” (cluster 2), “kidney development” and “sprouting angiogenesis” (cluster 6), “cellular catabolic process” (clusters 4 and 9), as well as processes typically found in the characteristic hypoxic and immune signature of ccRCC tumors^31^, such as “response to hypoxia” (cluster 8) and various immune-related processes (clusters 5, 7 and 10) (Fig. 1D and Supplementary Figure 1A). Most importantly, biological processes related to fibrosis, such as “extracellular matrix organization”, “extracellular structure organization” and “collagen metabolic process” were also found to be enriched in clusters 3 and 6 (Fig. 1D and Supplementary Figure 1A). Accordingly, we next performed histological assessments of original and CAM-xenografted ccRCC samples, using Masson’s trichrome staining, which revealed that variable collagen levels were found in tumors from ccRCC patients (Fig. 1E and Supplementary Figure 1B). Furthermore, the corresponding xenografts maintained similar abundance and patterns of intratumoral collagen fibers and/or extensive regions of fibrosis when cultivated on the CAM (Fig. 1E and Supplementary Figure 1B). Quantification of the percentage of collagen in tissues from 35 individual patients revealed a strong and significant correlation (r=0.8541; p<0.0001) between the percentage of collagen found in the original tumor and its corresponding CAM-xenografted ccRCC tissue (Fig. 1F).

**Fig. 2:**
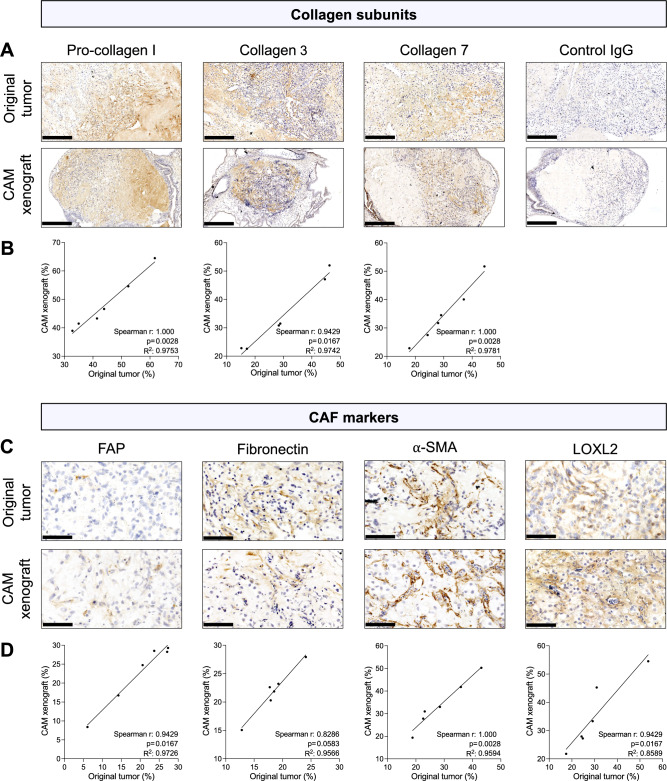
Clinically relevant fibrosis-associated markers in tumors from ccRCC patients are maintained on CAM. **A**-**D**) Collagen subunits (**A**-**B**) and CAF markers (**C**-**D**) in original tumor and CAM xenografts from patient #22-65. **A** and **C**) Representative immunohistochemistry images of collagen subunits (**A**) and CAF markers (**C**) (stained in brown, nuclei in blue) in ccRCC patient original and corresponding CAM xenografted tumors. Scale=250µm (**A**) or 50µm (**C**). B and **D**) Correlation between the percentage of positive areas for collagen subunits (**B**) or CAF markers (**D**) in the original and corresponding CAM xenografted tumors. Each dot represents the positive area for one patient (original tumor and associated PDXs from 6 patients), Spearman’s correlation test.

To further assess whether the xenografts grown on the CAM maintained the fibrotic stroma of ccRCC tumors, we selected a panel of fibril-associated collagens known to be involved in renal fibrosis (collagens type I, III, and VII)^32^ and performed immunohistochemical staining of both original and xenografted ccRCC tumors. Xenografts retained the collagen subunit expression pattern observed in the original patient tumor (Fig. 2A and Supplementary Figure 2), as shown by a strong correlation between the percentage of positive area for all markers in the original and the CAM xenografted tumors (Fig. 2B). To corroborate the importance of these collagen subunits in ccRCC prognosis, gene expression data from a TCGA cohort of 533 ccRCC patients were analyzed (KIRC-TCGA, available on the cbioportal.org database)^30^. Elevated expression of genes encoding for collagens type I, III, and VII was significantly associated with reduced 10-year overall survival in patients with ccRCC (*COL1A1*: HR=2.062, p=0.0005; *COL1A2*: HR=1.656, p=0.0162; *COL3A1*: HR=1.627, p=0.0235; *COL7A1*: HR=3.943, p<0.0001) (Supplementary Figure 3A). Because collagen deposition and cross-linking are tightly regulated by activated cancer-associated fibroblasts (CAF), we next assessed the expression of key CAF markers. In particular, ⍺-smooth muscle actin (⍺-SMA) and Fibroblast Activation Protein (FAP) were evaluated as indicators of myofibroblastic activation, while lysyl oxidase-like 2 (LOXL2) and fibronectin were examined for their role in collagen cross-linking and matrix stiffening, both processes linked to tumor progression^33^. Accordingly, original and xenografted ccRCC tumors from 6 individual patients were subjected to immunohistochemical staining for FAP, fibronectin, ⍺-SMA, and LOXL2. The expression of each of these markers was maintained in tumor fragments cultivated on the CAM, with levels strongly correlating to the percentage of positive area found in the patient’s original tumor (Fig. 2C-D and Supplementary Figure 2). Similarly to the collagen subunits, elevated expression levels of most CAF activation markers were also significantly associated with reduced overall survival (*FAP*: HR=2.150, p=0.0001; *FN1*: HR=1.554, p=0.0298; *ACTA2*: HR=0.9889, p=0.9568; *LOXL2*: HR=2.292, p<0.0001) (Supplementary Figure 3B). Taken together, these results indicate that key aspects of the fibrotic microenvironment of the original ccRCC tumors, were faithfully maintained in the CAM-ccRCC model.

### Response of human ccRCC tumors to antifibrotics can be measured using the CAM-ccRCC model

**Fig. 3:**
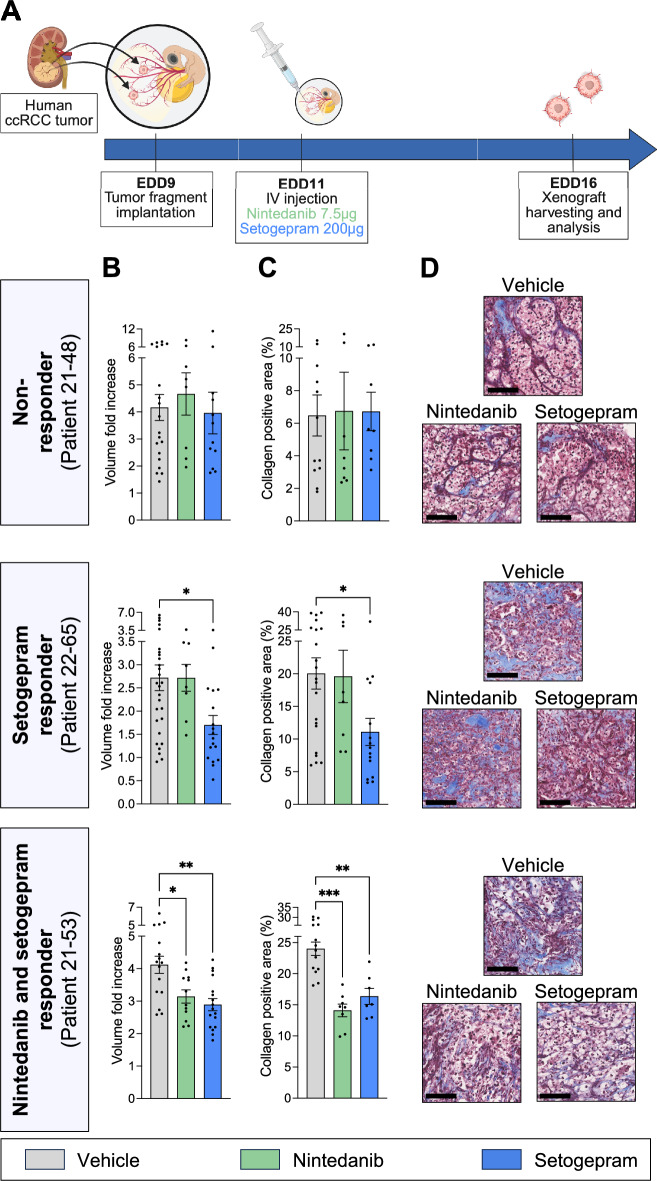
Differential patient response to antifibrotic therapies in the CAM-ccRCC model. **A**) Experimental protocol of antifibrotic treatments in the CAM-ccRCC model. Created in BioRender. Dubois, C. (2026) https://BioRender.com/b59g191. (**B-D**) Volume (**B**) and collagen positive area (**C**) with representative images (**D**) of tissue xenografts from 3 selected ccRCC patients (N=16 patients) following i.v. treatment with nintedanib (7.5 µg/embryo) or setogepram (200 µg/embryo), compared to vehicle. Each dot represents the result of one xenograft. Values are expressed as mean ± SEM. * P<0.05, ** P<0.01, *** P<0.001, Kruskal-Wallis test compared to vehicle. Scale=100µm.

To ascertain the suitability of the CAM-ccRCC model for evaluating the efficacy of antifibrotic agents in ccRCC tumors, nintedanib, an FDA-approved TKI for IPF treatment was selected, together with setogepram, a GPR84-targeted inverse agonist/antagonist previously demonstrated to diminish fibrosis in a CAM-IPF model^19^. Intravenous injections of doses comparable to those used in patients (7.5 µg nintedanib and 200 µg setogepram per embryo)^34,35^ or vehicle were administered at EDD11 (i.e. 2 days post implantation of the xenografts to allow perfusion of the xenografts by the CAM vasculature) (Fig. 3A). Xenografts were harvested at EDD16 and the volume and collagen content of CAM xenografts derived from 16 individual ccRCC patients were evaluated. Based on the reduction in collagen-positive area of xenografts treated with each of the antifibrotics, patients were classified into the following subgroups, non-responders (no significant response to any of the antifibrotic therapies) or responders (significant response to nintedanib and/or setogepram) (Table 2 and Fig. 3B-D). Of note, all (100%) patients exhibiting a decrease in collagen content post treatment with setogepram also presented a significant reduction in tumor volume, in contrast to only 50% for nintedanib, suggesting that setogepram response was principally attributed to collagen content reduction (Table 2).

**Table 2:** Response of ccRCC patient-derived xenografts to antifibrotic therapies.

**Response** **(collagen)**	**Patient**	**Nintedanib**		**Setogepram**	
		**% inhibition**		**% inhibition**	
		**Collagen**	**Volume**	**Collagen**	**Volume**
**Non-responders**	21-45	15.5	4.1	16.0	0
	21-48	0	0	0	4.9
	21-56	27.7	0	1.4	18.8
	22-62	0	26.4	13.8	24.5
	22-63	0	3.1	0	0
	22-68	3.9	**37.7**	15.4	7.6
	22-69	0	0.8	0	0
**Responders**	21-47	**36.0**	**65.9**	20.7	16.2
	21-54	**31.5**	**37.5**	16.5	8.0
	21-58	0	**28.3**	**62.7**	**58.7**
	22-60	18.9	0	**36.6**	**33.2**
	22-64	0	**32.0**	**61.6**	**40.3**
	22-65	2.2	0.1	**44.7**	**37.4**
	22-70	17.4	**30.7**	**41.9**	**21.0**
	21-53	**41.3**	**23.7**	**31.8**	**29.8**
	22-67	**52.6**	**52.8**	**50.0**	**34.5**
**Concordance collagen/volume**		**4/8 (50%)**		**7/7 (100%)**	

Bold values: Significant responses (P<0.05), Wilcoxon-Mann-Whitney test compared to vehicle.

**Fig. 4:**
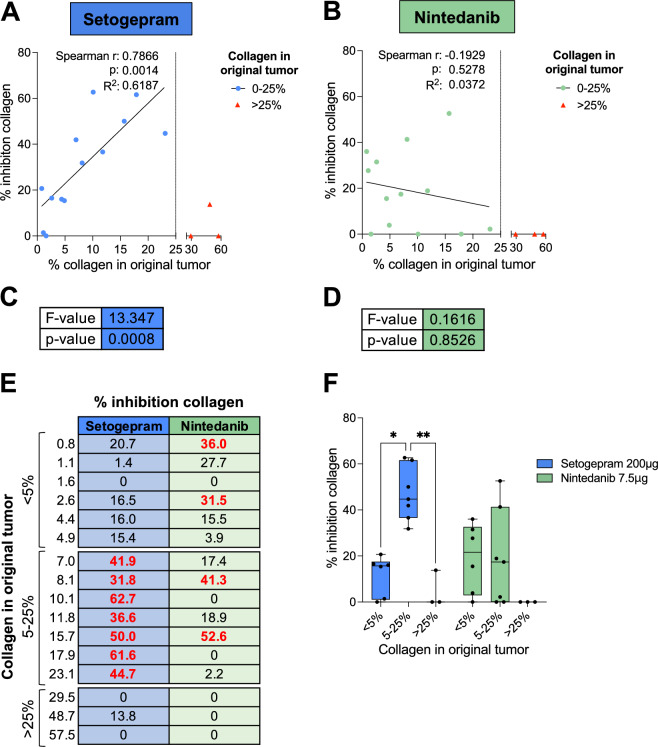
Response to antifibrotic therapies according to the collagen content of original patient tumors. (**A** and **B**) Correlation between the collagen content of original tumors and the percentage of inhibition of collagen in corresponding CAM xenografted tumors (N=16 patients) following i.v. treatment with setogepram (200 µg/embryo) (**A**) or nintedanib (7.5 µg/embryo) (**B**). Each dot represents the result for one patient, Spearman’s correlation test. (**C** and **D**) Summary of the statistical significance between the the simple linear model containing all patients and the segmented model with a breakpoint at x=25 for xenografts treated with setogepram (**C**) or nintedanib (**D**). ANOVA for nested model comparing using an F-test. E and F) Representation of the significant responses (**E**) (Red=Significant responses (p<0.05), Wilcoxon-Mann-Whitney test compared to vehicle) in the percentage of collagen inhibition of xenografts from ccRCC patients with <5%, 5-25% and >25% collagen levels in their original tumor (N=16 patients) following i.v. treatment with nintedanib (7.5 µg/embryo) or setogepram (200 µg/embryo) (**F**). Each dot represents the result of one xenograft. Values are expressed as mean ± SEM. * P<0.05, ** P<0.01, Kruskal-Wallis test compared to 5-25% collagen.

Following the observation that different patients respond differentially to the antifibrotics in this model, we investigated whether the collagen content of the patient’s original tumor could predict the response to antifibrotic therapy. The antifibrotic response to setogepram, measured as the percentage inhibition of collagen content in ccRCC CAM xenograft, was significantly correlated with the collagen levels in the original tumors for cases with less than 25% intratumoral collagen (p=0.0014) (Fig. 4A). However, no such correlation was observed for nintedanib (p=0.5278) (Fig. 4B). To better capture potential non-linear relationships and account for the biological heterogeneity between low- and high-fibrosis tumors, we applied segmented linear regression models with a fixed breakpoint at 25% collagen in original tumors. This approach allows the identification of variations in drug sensitivity profiles in tumors displaying different levels of initial fibrosis, as opposed to assuming a uniform relationship across the entire range of collagen content. For setogepram, the segmented model provided a significantly better fit compared to the simple linear model (F=13.347, p=0.0008), highlighting a fibrosis-dependent drug effect (Fig. 4C). In contrast, no improvement was observed for nintedanib (F=0.1616, p=0.8526), suggesting that its activity was not influenced by baseline fibrosis (Fig. 4D). According to these observations, patients were initially stratified into two subgroups based on collagen content in their original tumors (<25% vs >25%) and response to setogepram. However, we found that only a smaller subset of patients (5-25% initial collagen content) significantly responded to this antifibrotic (Fig. 4E). To more precisely capture this pattern of response, we refined the stratification into three subgroups: low (<5%), intermediate (5-25%) and high (>25) collagen levels. When compared to the intermediate group, xenografts from patients with either very low (<5%) or high (>25) collagen in their original tumor exhibited significantly lower percentage of collagen inhibition following treatment, highlighting the intermediate collagen group as the most sensitive to setogepram (Fig. 4F). Additionally, tumors from patients classified as responders exhibited significantly greater GPR84 expression compared to non-responders (Supplementary Figure 4), which is in line with the mechanism of action of this GPR84-targeting agent. Together, these findings demonstrate that the CAM-ccRCC model is suitable for assessing antifibrotic responses and suggest that tumors with intermediate collagen levels (5–25%) are most likely to benefit from such therapies, with maximal efficacy observed in this fibrosis range.

### Reducing fibrosis as a therapeutic approach to ameliorate response to sunitinib in ccRCC tumors

**Fig. 5:**
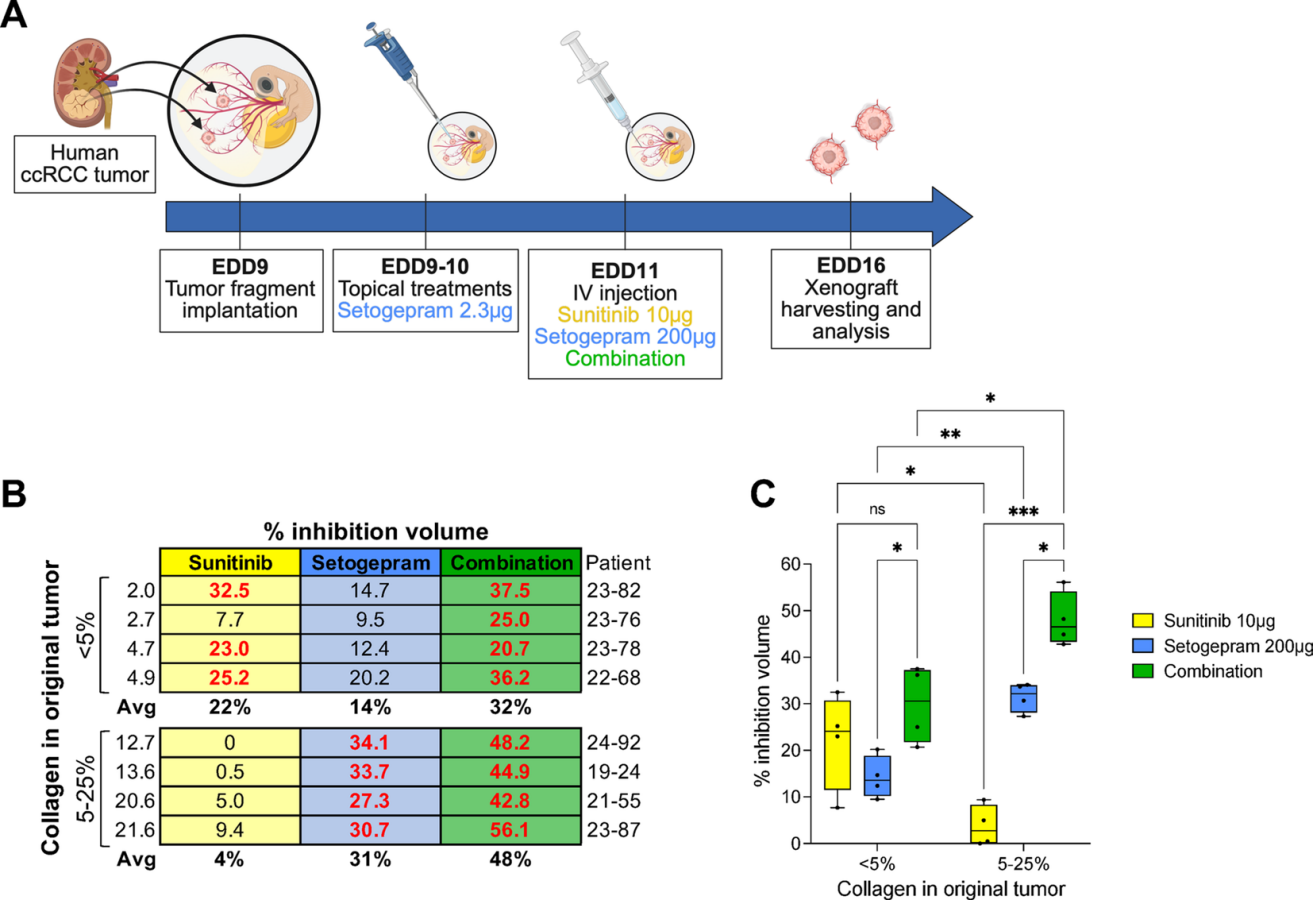
ccRCC xenografts from tumors with 5-25% collagen have a better response to setogepram and sunitinib co-therapy. **A**) Experimental protocol of co-therapy in the CAM-ccRCC model. Created in BioRender. Dubois, C. (2026) https://BioRender.com/garxyv9. **B** and** C**) Representation of the significant responses (**B**) (Red=Significant responses (p<0.05), Kruskal-Wallis test compared to vehicle) in the percentage of volume inhibition of xenografts from ccRCC patients with <5% and 5-25% collagen levels in their original tumor (N=8 patients) following i.v. treatment with sunitinib (10 µg/embryo) or pretreatment with setogepram (2.3 µg/xenograft) 2 days prior i.v. treatment with setogepram (200 µg/embryo) or a combination therapy (sunitinib 10 µg and setogepram 200 µg/embryo) (**C**). Avg: Average of percentage of inhibition of volume for each treatment. Each dot represents the mean percentage volume inhibition of all xenografts for each individual donor (N=8 patients). Values are expressed as mean ± SEM. Two-way ANOVA with Tukey’s multiple comparison test.

Resistance to therapy remains a major clinical challenge for ccRCC patients, with increasing evidence indicating that cancer-associated fibrosis can influence the sensitivity of cancer cells to treatment^9,10^. Building on our observation that intratumoral fibrosis is regulated in the CAM-ccRCC model, we next hypothesized that therapeutic response to antitumoral agents is linked to modulation of intratumoral fibrosis. As setogepram emerged as the most effective antifibrotic treatment in the CAM-ccRCC model, we selected this compound to evaluate the therapeutic potential of antifibrotics in improving the response to sunitinib, a TKI approved for metastatic ccRCC^36^. For this purpose, we focused on tumor samples with low <5% and intermediate 5-25% collagen, as these subgroups showed a significant correlation between the collagen content in original tumors and the response to setogepram in the CAM assay (Fig. 4A). Moreover, these two groups represented the majority of patients in the Sherbrooke ccRCC cohort (86% of patients), while only 14% had >25% collagen. To decrease intratumoral fibrosis, ccRCC-derived xenografts received a pretreatment of 2.3µg setogepram per xenograft for two days prior to sunitinib administration (10µg/CAM) alone or in combination with setogepram (200µg/CAM) (Fig. 5A). The findings indicate that the combination therapy of setogepram and sunitinib was significantly more effective (48% inhibition, p=0.0007) than sunitinib monotherapy (4% inhibition) to reduce the volume of ccRCC xenografts in the 5-25% collagen subgroup (Fig. 5B and C). Furthermore, the mean decrease in volume with sunitinib monotherapy was considerably higher (p=0.0310) in xenografts obtained from patients in the <5% collagen subgroup compared to those in the 5-25% collagen subgroup (22% versus 4% inhibition, respectively). These results are consistent with the notion of increased drug sensitivity in tumors with minimal or reduced fibrosis.

**Fig. 6:**
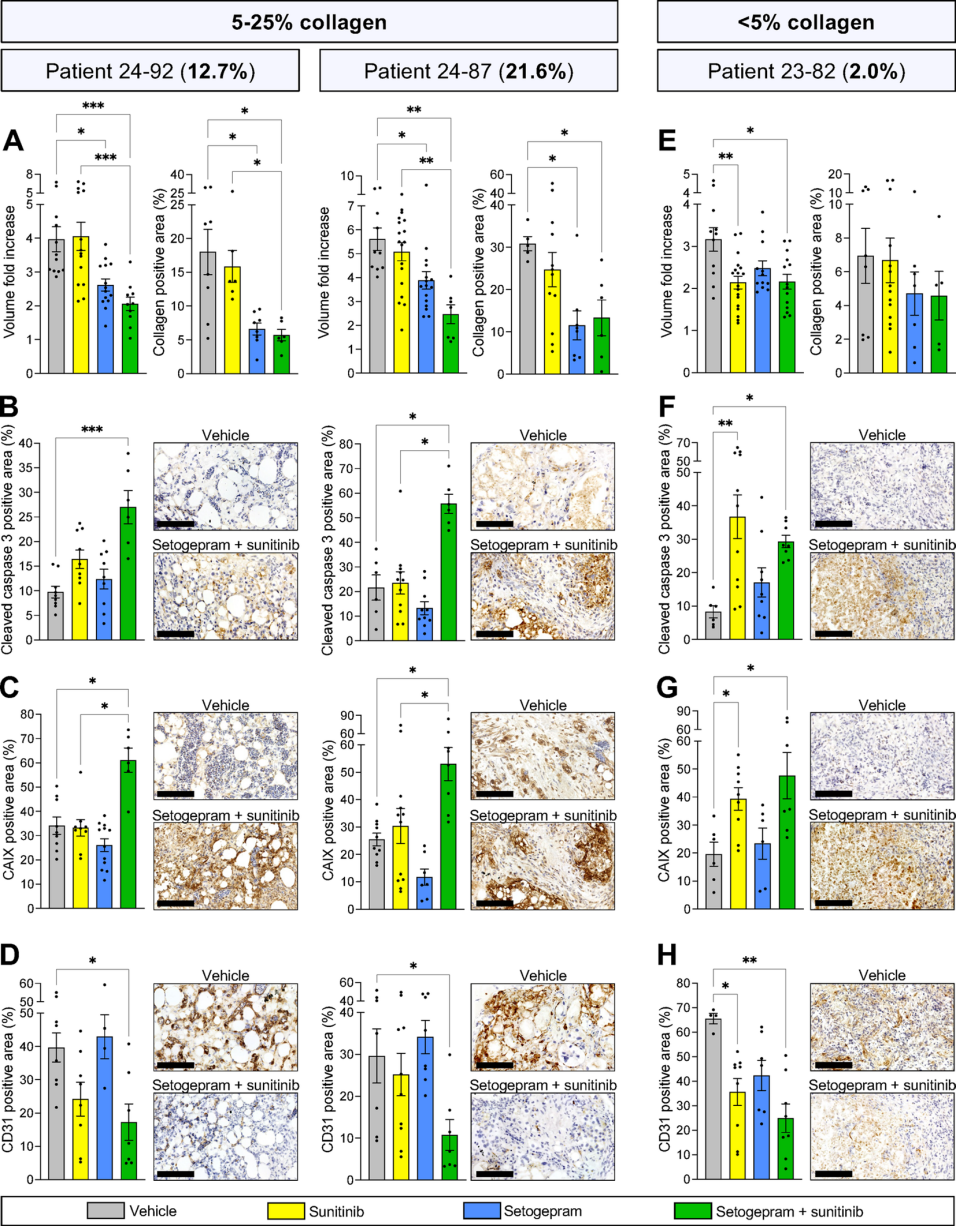
Setogepram potentiates the response to sunitinib in ccRCC-derived xenografts with 5-25% initial collagen content. (**A**-**H**) Xenografts from ccRCC patients with 5-25% (**A**-**D**) and <5% (**E**-**H**) collagen levels in their original tumor were treated with sunitinib (10 µg/embryo i.v.) or pretreated with setogepram (2.3 µg/xenograft) 2 days prior to i.v. treatment with setogepram (200 µg/embryo) or a combination therapy (sunitinib 10 µg and setogepram 200 µg/embryo). Response of xenografts was measured by modulations in volume and collagen positive area (**A** and **E**), cleaved caspase 3 (**B** and **F**), CAIX (**C** and **G**) or CD31 (**D** and **H**) positive area, compared with vehicle or sunitinib monotherapy. Representative immunohistochemistry images are shown (stained in brown, nuclei in blue), (N=3 patients). Scale=100µm. Each dot represents the result of one xenograft. Values are expressed as mean ± SEM. * P<0.05, ** P<0.01, *** P<0.001, Kruskal-Wallis test compared with vehicle.

Sunitinib is known to mediate its antitumor effects by reducing angiogenesis through vascular endothelial growth factor receptor (VEGFR)1, 2, 3 and platelet-derived growth factor receptor (PDGFR)β inhibition, which results in reduced oxygen supply to cancer cells and thereby upregulation of hypoxia and apoptosis^37^. To confirm that pretreatment with setogepram enhanced response to sunitinib in the antifibrotic-sensitive 5-25% collagen subgroup (Fig. 6A), we performed immunohistochemistry for cleaved-caspase 3 (apoptosis), CAIX (hypoxia) and CD31 (blood vessels)^38^. Sunitinib alone failed to induce any significant changes, whereas the combination therapy strongly modulated key antiangiogenic drug response indicators, i.e. increased cleaved-caspase 3 and CAIX and reduced CD31, suggesting enhanced intratumoral activity of sunitinib (Fig. 6B-D). As a control, in xenografts from a patient in the sunitinib-sensitive low <5% collagen subgroup (Fig. 6E), sunitinib alone significantly increased cleaved-caspase 3 and CAIX, and decreased CD31, with no additional effect when combined with setogepram (Fig. 6F-H).

Collectively, these findings indicate that targeting fibrosis using antifibrotics may enhance TKI efficacy in ccRCC, especially in tumors characterized by intermediate (5-25%) collagen content.

## Discussion

The fibrotic stroma of ccRCC tumors drives tumor progression and resistance to therapy. Unfortunately, the modest efficacy of current therapeutic targets for intratumoral fibrosis, coupled with a deficiency of robust preclinical models, hinders the identification of new effective therapies. In this study, we show that the CAM-ccRCC model faithfully preserves collagen, fibrosis-associated markers, and key pathways enriched in tumors from ccRCC patients. In addition, we observed that treatment with antifibrotic agents can effectively modulate intratumoral fibrosis in ccRCC xenografts, and that collagen content in the original tumors can predict the response to antifibrotic therapy. Moreover, the antifibrotic setogepram improved the antitumor efficacy of the TKI sunitinib, with the most pronounced benefit observed in the highly responsive subgroup of tumors with an intermediate 5-25% level of collagen.

A significant limitation of current preclinical models to accurately evaluate the clinical potential of antitumor drugs is largely due to their inability to recapitulate or preserve the integrity of the human fibrotic tumor microenvironment, a critical determinant of tumor progression and resistance to therapies due to its impact on drug bioavailability, angiogenesis, and metastasis^10^. In fact, the study of human stroma mostly relies on *in vitro* co-culture models of cancer cells and fibroblasts, however they lack many stromal cell types and ECM components to fully capture the dynamic interplay and heterogeneity characteristic of a real tumor^39^. More complete *in vivo* models have been developed by growing tumors using cell lines or PDXs in immunocompromised mice^39^. However, the suboptimal engraftment rate of xenografts in murine models often requires multiple amplification cycles to obtain adequate tumor samples, a procedure that leads to the replacement of the patient’s tumor microenvironment with the host’s stroma^40^. Our findings demonstrate that the human stromal microenvironment, including fibrosis-associated collagen subunits and CAF markers, is preserved in ccRCC-derived xenografts grown on the CAM for 7 days, exhibiting comparable levels and composition to the original tumor. This was further supported by PCA and heatmap analyses of transcriptomic data from patient samples, showing that CAM xenografts retain the expression profile of their original tumor, which is markedly distinct from the CAM (host) tissue. Furthermore, GO analysis of the 2,500 most highly modulated transcripts in the original tumors and CAM xenografts identified two extracellular matrix-related clusters (clusters 3 and 6) preserved in the tumor and CAM xenografts, but absent in the CAM sample. This retention of the human stromal signature is of particular interest, as previous gene expression analyses of ccRCC tumorgrafts grown in immunodeficient mice have shown an underrepresentation of transcripts encoding human stroma, due to its extensive replacement by murine stroma^41^. This distinctive advantage of the CAM-ccRCC model makes it particularly well-suited for assessing the clinical potential of antifibrotic therapies and for the development of optimal drug candidates to treat these challenging fibrotic tumors. In this context, future optimization of the model could incorporate additional ECM-related parameters to further enhance its predictive accuracy, including the quantification of total hydroxyproline levels and comprehensive assessment of collagen content and structural organization across whole xenografts, thereby complementing the tissue section-based histological analyses presented in this study. Moreover, a deeper characterization of temporal stromal dynamics during the 7-day period of xenograft cultivation on the CAM would be valuable to determine whether fibroblasts, as well as other populations such as immune cells, expand over time and whether collagen composition evolves dynamically. However, such analyses are inherently constrained by the relatively short experimental timeframe of the CAM model, which may limit the ability to capture longer-term processes of fibrosis remodeling and treatment response.

Novel TKI, mTOR pathway-, and immune checkpoint-directed therapies have demonstrated efficacy in ccRCC, but their effectiveness is difficult to predict, which translates to poor overall outcomes^7^. To address this, current research focuses on identifying biomarkers and evaluating new therapeutic approaches. Using the CAM-ccRCC model, we investigated whether intratumoral collagen content could help predict response to antifibrotics in ccRCC patients. The efficacy of setogepram in reducing collagen levels was found to be dependent on the initial collagen burden of the parental tumor, with a significant reduction observed in xenografts from tumors with an initial collagen content between 5-25%. Consistent with the mechanism of action of this GPR84-targeting agent, tumors from this patient group displayed significantly higher GPR84 expression than non-responders, suggesting a plausible biological basis for treatment sensitivity. In fact, this receptor is known to be expressed in multiple cell types involved in fibrotic processes, including immune cells, fibroblasts, and epithelial cells^42^. These cell populations have individually been reported to respond to setogepram^43^. While further studies will be required to delineate the relative contribution of each cell type to the therapeutic response observed in our model, this pleiotropic expression pattern may help explain the enhanced antifibrotic response observed in xenografts from the high-GPR84-expressing subgroup of patients. On the other hand, xenografts derived from tumors with a lower (<5%) and higher (>25%) initial collagen burden showed no reduction in collagen levels in response to this antifibrotic. The lack of response to antifibrotics in these two groups may reflect distinct limitations: in the low collagen subgroup, the minimal degree of fibrosis may be insufficient to allow measurable modulation, whereas in highly fibrotic tissues, the dense extracellular matrix may hinder drug penetration, and the reduced pool of treatment-responsive stromal cells further limits antifibrotic efficacy^44,45^. In contrast, nintedanib, although an approved antifibrotic therapy for IPF^13^, demonstrated limited efficacy in our model: only 4 of 16 patient-derived xenografts showed a significant reduction in collagen, with responders dispersed across both <5% and 5–25% collagen subgroups. This restricted activity is likely due to its mechanism of action. Nintedanib targets VEGFR, PDGFR and FGFR, receptor tyrosine kinases that overlap with those inhibited by sunitinib and other TKIs already in used in ccRCC, therapies known to display modest efficacy and resistance when used as monotherapy^46,47^. These results underscore that not all antifibrotic agents provide predictable or collagen-dependent effects in ccRCC, highlighting the need for biomarkers capable of discerning which patients are likely to benefit from antifibrotic strategies. Given that the 2024 ESMO Clinical Practice Guideline mandates histopathological confirmation of RCC for all patients before systemic treatment is initiated, such analysis of intratumoral collagen could likely be performed on existing patient biopsies^48^.

By faithfully preserving the fibrotic stroma, the CAM ccRCC model allows us to directly link intratumoral collagen content to treatment response in patient-derived ccRCC tumors. This is particularly relevant given that stromal heterogeneity is increasingly recognized as a determinant of therapeutic outcome across solid tumors^49,50^, and specifically in ccRCC^9,51^. In our analysis, tumors with low fibrosis (<5% collagen) exhibited the strongest response to the antiangiogenic drug sunitinib, that includes increased apoptosis and hypoxia, supporting the concept that low stromal density facilitates drug distribution and limits the establishment of hypoxic niches^52,53^. In contrast, in tumors with intermediate fibrosis (5–25%), pharmacological ECM remodeling with setogepram significantly improved sunitinib efficacy, highlighting the potential of pharmacological stroma modulation to overcome intrinsic resistance mechanisms. These observations are consistent with findings from other cancer models, where ECM accumulation has been shown to promote hypoxia, reduce drug efficacy, and impede immune cell infiltration into the tumor microenvironment^54–56^. Such parallels reinforce the idea that fibrosis is not merely a passive histological feature, but an active contributor to therapeutic resistance in ccRCC. Importantly, our data derived from stromal-preserved PDX models support the emerging concept of stromal precision medicine^50^, in which patients could be stratified according to fibrosis levels to optimize the use of TKIs, either as monotherapy in low-fibrosis tumors or in combination with stroma-targeting agents in tumors with higher fibrotic content. Furthermore, as intratumoral fibrosis is known to promote immune cell exhaustion and impede their infiltration into the tumor microenvironment, reducing fibrosis using antifibrotic agents could also enhance the efficacy of immunotherapies currently used for ccRCC patients^10,57^. However, as these threshold for therapeutic stratification remain hypothesis-generating, additional research involving validation in independent and larger cohorts will be important to establish their robustness and broader applicability. Such studies will also be critical to confirm the clinical utility of collagen thresholds as predictive biomarkers and to clarify the mechanisms by which ECM modulation improves treatment response in ccRCC.

In conclusion, our study identified the CAM-ccRCC model as a valuable platform for evaluating the potential of antifibrotic strategies to reverse the fibrotic stromal signature of ccRCC tumors and improve the efficacy of anticancer therapies. It also lays the groundwork for the use of personalized therapeutic models that integrate fibrotic stroma-dependent determinants of drug sensitivity in ccRCC.

## Supplementary Information

Supplementary Information.

## Data Availability

All data generated or analyzed during this study are included in this published article and its supplementary information files or from the corresponding author on reasonable request. The datasets generated and analysed during the current study are available in the Gene Expression Omnibus (GEO) repository (GSE315653).
