# Supplementary material for: A patient-derived ccRCC model that retains native stromal features to assess fibrosis-driven personalized therapeutic response
Source: Sci Rep. 2026 Apr 28;16:19713. doi: 10.1038/s41598-026-50508-z (PMC13315207; doi:10.1038/s41598-026-50508-z)
Supplement: Supplementary file 1 — Supplementary Information. [file 41598_2026_50508_MOESM1_ESM.docx]

**Supplementary Information**

**A Patient-Derived ccRCC Model That Retains Native Stromal Features to Assess Fibrosis-Driven Personalized Therapeutic Response**

**Alexis Perreault**^1^**, Kelly Harper**^1^**, Martine Charbonneau**^1^**, Karine Brochu-Gaudreau**^1^**, Robert Sabbagh**^2^**, Patrick O. Richard**^2^**, Nadia Ekindi-Ndongo**^3^**, Claudio Jeldres**^2^**, Claire M. Dubois**^1*^

^1^Department of Immunology and Cell Biology, Université de Sherbrooke, Sherbrooke, J1H 5N4, QC, Canada.

^2^Division of Urology, Department of Surgery, Université de Sherbrooke, Sherbrooke, J1H 5N4, QC, Canada.

^3^Department of Pathology, Université de Sherbrooke, Sherbrooke, J1H 5N4, QC, Canada.

*Corresponding author

Email: [Claire.Dubois@USherbrooke.ca](mailto:Claire.Dubois@USherbrooke.ca)

**Supplementary figure 1**. Relevant gene clusters and collagen levels in tumors from ccRCC patients are maintained in CAM PDX.

**A**) GO enrichment dot plots for genes in clusters 1-5 and 7-10. Adjusted p-values <0.05 using Benjamini-Hochberg method for False Discovery Rate correction. **B)** Representative Masson’s trichrome staining images of the patient’s original tumor compared to the corresponding CAM xenograft (original tumor and associated PDXs from 2 patients), showing collagen deposition patterns (collagen in blue, cytoplasm in red, nuclei in black). Scale=1mm (patient tumor), 250µm (CAM xenograft) and 50µm for higher magnifications.

**Supplementary figure 2**. Fibrosis-associated markers in tumors from ccRCC patients are maintained on CAM.

Representative immunohistochemistry images of collagen subunits and CAF markers (stained in brown, nuclei in blue) in ccRCC patient original and corresponding CAM xenografted tumors (original tumor and associated PDXs from 2 patients). Scale=250µm (collagen subunits) or 50µm (CAF markers).

**Supplementary figure 3**. Fibrosis-associated markers maintained on CAM are associated with ccRCC patient survival.

**A and B**) Kaplan-Meier survival curves of 10-year overall survival of ccRCC patients according to the gene expression of collagen subunits (**A**) or CAF markers (**B**). HR=Hazard ratio (logrank test) high expression/low expression. P was calculated using the Logrank test (Mantel-Cox) (KIRC-TCGA cohort (N=533).

**Supplementary figure 4**. GPR84 levels in ccRCC tumors are associated with response to setogepram.

The GPR84-positive region within the initial tumors of patients who exhibited either a significant (responders) or a non-significant (non-responders) decrease in collagen content after intravenous setogepram treatment (200 µg/embryo) of CAM xenografts. Each dot represents the percentage of GPR84-positive area in the original tumor of one ccRCC patient (N=23 patients). Values are expressed as mean ± SEM. *** P<0.0001, Wilcoxon-Mann-Whitney test.
